# The role of red blood cell exchange for severe imported malaria in the artesunate era: a retrospective cohort study in a referral centre

**DOI:** 10.1186/s12936-016-1264-z

**Published:** 2016-04-14

**Authors:** Antonia Calvo-Cano, Joan Gómez-Junyent, Miguel Lozano, Pedro Castro, Joan Cid, Jose María Nicolás, Llorenç Quintó, Maite Martin, Jose Muñoz, Joaquim Gascon

**Affiliations:** ISGlobal, Barcelona Ctr. Int. Health Res. (CRESIB), Hospital Clínic-Universitat de Barcelona, Barcelona, Spain; Apheresis Unit, Department of Hemotherapy and Hemostasis, CDB, IDIBAPS, Hospital Clínic, University of Barcelona, Barcelona, Spain; Intensive Care Unit, Hospital Clínic, Barcelona, Spain; Pharmacy Department, Hospital Clínic, Barcelona, Spain

**Keywords:** Malaria, Severe malaria, *Plasmodium falciparum*, Automated red blood cell exchange, Artesunate

## Abstract

**Background:**

Intravenous artesunate has replaced quinine as the first-line therapy for severe imported malaria, given its anti-malarial superiority shown in clinical trials conducted in endemic countries. Evidence for red blood cell (RBC) exchange in patients with severe malaria treated with artesunate is lacking. This retrospective cohort study describes the experience at Hospital Clinic of Barcelona with the use of artesunate for severe malaria and the joint use of RBC exchange in selected cases.

**Methods:**

Patients treated for severe malaria at Hospital Clinic of Barcelona between August 2013 and January 2015 were included in this retrospective study. Severe malaria was defined according to WHO criteria. Data were extracted from electronic hospital records. A log-linear mixed model approach was used to estimate parasite clearance times.

**Results:**

Within the study period, 42 patients were diagnosed of malaria at this centre, of which 38 had *Plasmodium falciparum* (90.5 %). Sixteen patients (42 %) had severe malaria cases and were treated with intravenous artesunate. Four patients underwent RBC exchange within a period of 15 h after the first dose of artesunate (range 9–21 h). The procedure lasted a median of 2 h (IQR 1.8–2 h), using a median of 12 (IQR 11–14) units of packed RBCs to replace a median of 3794 ml (IQR 2977–4343). The technique was well-tolerated without haemodynamic complications. There were no deaths. The regression model showed an estimated time to 95 % decay of 21.6 h (95 % CI 17.3–28.8). When assessing effect modification by RBC exchange, there was no difference in the parasite elimination rate (p = 0.286).

**Discussion and conclusion:**

In this study RBC exchange failed to show benefits in terms of parasite clearance probably due to the small number of patients analysed. The evidence for exchange transfusion remains limited.

## Background

The management of severe imported malaria remains a challenge of consensus, since the elaboration of clinical guidelines always has to deal with the lack of evidence in non-endemic countries. The design of clinical trials randomizing cases of severe imported malaria is unrealistic nowadays, due to the high number of subjects required, the heterogeneous group of affected travellers in terms of parasitaemia and clinical presentation/complications., and ethical problems given the evidence from endemic regions [1, 2]. The last and most important change in guidelines of imported severe malaria is the replacement of quinine by intravenous artesunate (IVA) as the first-line therapy [3–5]. Superior efficacy and reduction of mortality have been shown in clinical trials in Asia and Africa [6, 7] and confirmed in European retrospective studies [8]. Despite international consensus in using IVA for severe malaria, it is not yet available as a licensed drug in all non-endemic countries. So far, it is ordered as compassionate use or as investigational new drug protocol. In this context, it is difficult to collect data to provide evidence of the safety profile or anti-malarial efficacy. Regarding safety profile, some post-introduction data have suggested delayed haemolytic anaemia as an adverse event that should be monitored the next 4 weeks after IVA [9–11]. Better data is needed in terms of parasite response, which is commonly measured through the parasite clearance time (PCT). Although parasite clearance has not been validated as a surrogate endpoint for death from severe malaria, it is an accepted marker of treatment response. PCT50 is defined as the time required to reduce the initial parasitaemia by 50 %, under anti-malarial treatment. Improvement of PCT with IVA versus quinine [5–7] poses a new scenario that questions the role of some adjunctive therapies, such as exchange transfusion and red blood cell (RBC) exchange.

Different strategies have been proposed as adjunctive therapies, mainly helping pharmacologic treatment to reduce parasitaemia. Whole blood exchange transfusion has been suggested and used with this purpose. Its theoretical benefits are based on the removal of: (i) infected circulating red blood cells (RBCs) (those sequestered in microvasculature are not affected); (ii) rigid non-infected RBCs, with a rheological effect; (iii) plasma toxins, minimizing systemic inflammatory response and sequestration. It has demonstrated a rapidly reduction in parasite biomass [12], but has not shown effectiveness to improve survival [13]. RBC exchange has been advocated as a better alternative to whole blood exchange transfusion in the adjunctive treatment of severe imported malaria [14–16]. RBC exchange does not remove plasma toxins, but has significant advantages over exchange transfusion in terms of speed, efficiency, haemodynamic stability and retention of plasma components such as clotting factors and may thus represent an improvement in adjunctive therapy for severe malaria [17]. Although RBC exchange it is recommended by some national apheresis societies [18], conclusive evidence is lacking. Some case reports and patient cohorts have been reported, but mainly in conjunction with quinine [14, 19], or using exchange transfusion or RBC exchange [20].

This study reports our experience with the use of artesunate for treating severe malaria and the use of RBC exchange in selected cases treated at Hospital Clinic of Barcelona in Spain between August 2013 and January 2015.

## Methods

### Study setting and design

This retrospective cohort study includes all patients with severe malaria treated with intravenous artesunate at Hospital Clínic, in Barcelona, Spain, between August 2013 and January 2015. Hospital Clínic is a national reference centre for Tropical Imported Diseases. In 2013, artesunate became available in Hospital Clínic for the treatment of patients with severe malaria. Patients with severe malaria are initially managed in the intensive care unit and subsequently, when they have improved, transferred to a conventional ward.

### Study procedures

Patients were diagnosed with severe malaria if their thick or thin blood film showed asexual stages of *Plasmodium falciparum* and fulfilled one or more WHO criteria (Table 1). Parasite counts were calculated as a percentage of the number of parasitized RBC in a thin blood film. Blood films were performed during the clinical management of the patient when considered by the treating physician and until no parasites were observed.

**Table 1:** Severity criteria in *Plasmodium falciparum* malaria

Type	Criteria	Definition
Clinical	Impaired consciousness	Any impairment in consciousness, not related to other causes (hypoglycaemia, concomitant infection)
	Prostration	Generalized weakness so that the patient is unable to sit, stand or walk without assistance
	Multiple convulsions	More than two episodes within 24 h
	Respiratory failure	PaO_2_ <60 mmH (FiO2 21 %)
	Shock	Systolic blood pressure <80 mmHg
	Jaundice	Clinical jaundice plus evidence of other vital organ dysfunction
	Bleeding	Spontaneous bleeding without any other potential cause
Analytical and radiological	Hypoglycaemia	<2.2 mmol/l or <40 mg/dl
	Metabolic acidosis	Plasma bicarbonate <15 mmol/l
	Severe normocytic anaemia	Haemoglobin <5 g/dl
	Haemoglobinuria	Haemoglobin in urine
	Hyperlactataemia	Lactate >5 mmol/l
	Renal impairment	Serum creatinine >265 µmol/l
	Pulmonary oedema or distress respiratory syndrome	Bilateral alveolar infiltrates in chest x-ray
Parasitological	Hyperparasitaemia	Parasitaemia >2.5 %

Adapted from World Health Organization [2]

Intravenous artesunate was administered at doses of 2.4 mg/kg at 0, 12, 24 and then every 24 h until parasitaemia was cleared. Intravenous artesunate was followed by a course of oral atovaquone-proguanil (1000/400 mg) during 3 days.

RBC exchange was performed at the decision of the treating physician in conjunction with Haemotherapy specialists. In general, it was considered as an adjunctive treatment in patients who had high baseline parasitaemia (generally above 30 %) and shock, respiratory failure or cerebral malaria. RBC exchange was also performed if patients were severely ill and parasitaemia did not decrease at least 25 % compared to baseline after 8–12 h of artesunate. RBC exchanges were performed in a COBE Spectra blood separator (TerumoBCT, Lakewood, Co, USA) using the RBCX software. Sex, height, weight, patient’s haematocrit, average haematocrit in the replacement red blood cells units, desired end haematocrit for the patient, fluid balance and the fraction of infected RBC remaining at the end were introduced at the start of the procedure. Once the initial data is entered, the replacement volume of RBC to be exchanged proposed by the COBE Spectra software was followed. ACD-A was used as anticoagulant at a infusion rate of 1.2 mL/min/L total blood volume with prophylactic calcium and magnesium infusion administration [21]. Fresh leukoreduced RBC units in SAG-Mannitol additive solution were used as the replacement solution. If possible, peripheral vein accesses were used for the procedure. If not, a double lumen catheter was inserted in the internal jugular vein.

### Data collection

All patients included into the study were identified prospectively in the clinical routine practice. A retrospective review of their electronic records was performed and an anonymized case report form was filled out. This included the following data: demographics and travel history; WHO clinical, analytical and radiological severity criteria; dates and times on symptoms initiation, hospital attendance, blood films performed and treatment commencement; the number of artesunate doses and other antimicrobials previously administered; complications and mortality. Data on RBC exchange was provided by the Department of Hemotherapy and Hemostasis, which keeps a registry of the apheresis performed in the hospital.

### Statistical analysis

The data collected were entered into Epi Info 7 (CDC, Atlanta, USA) and then transferred to Stata 13.1 (Stata Corporation, Texas, USA) for the analysis. Categorical variables were described by counts and percentages, whereas median and interquartile ranges (IQR) were used to summarize continuous variables. Comparisons between groups were performed with Fisher exact test and Mann–Whitney test for categorical and continuous variables, respectively.

Parasite clearance times (PCT) were estimated since the administration of the first dose of artesunate. Initially, the parasite loads were calculated from the parasite counts expressed in percentages and the number of RBC per microlitre in each blood film. The variation of parasite loads was analysed using a log-linear mixed-effects regression model incorporating a Gaussian random intercept. This resulted in an estimation of the dynamics of parasitaemia, assuming a single exponential model, allowing calculating the decrease of log parasitaemia per unit of time (hour) or parasite elimination rate. The interaction between RBC exchange and time was included in the model to evaluate the possible modification of the effect of artesunate in clearing parasitaemia.

The average reduction rate per hour was obtained based on the coefficient of the regression model (1- exponential of the coefficient of regression), as well as its 95 % confidence interval. The model estimates obtained were used to calculate the time to 50 % (PCT50 %), 75 % (PCT75 %), 90 % (PCT90 %), 95 % (PCT95 %) and 99 % (PCT99 %) clearance of parasitaemia. 95 % confidence intervals (95 % CI) and p values were obtained for all estimates in the model.

### Ethical considerations

The Ethics Committee of Hospital Clínic approved this study. Data collection forms were completely anonymous. As it was a retrospective study, the Ethics Committee approved waiver of individual consent from patients whose routine data was used.

## Results

### Patients’ baseline characteristics

From August 2013 to January 2015, 42 patients presented with malaria, 38 by *P. falciparum* (90.5 %), and 16 of those (42.11 %) were identified as severe malaria cases. All 16 were treated with IVA, and 4 additionally with RBC exchange. Their baseline characteristics are summarized and compared in Table 2.

**Table 2:** Characteristics of 16 patients with severe malaria treated with artesunate with or without RBC exchange

	Overall (n = 16)	Artesunate only (n = 12)	Artesunate and RBC exchange (n = 4)	p value
Demographics				
Age	40 (33–50)	36 (32–48)	47 (39–55)	0.332
Male	11 (68.75)	8 (66.67)	3 (75)	0.755
Clinical data				
Number of severity criteria	3 (1–6)	1 (1–5)	7 (6–10)	0.016
Clinical criteria				
>2 criteria	6 (37.5)	3 (25)	3 (75)	0.074
Impaired consciousness	3 (18.75)	2 (16.67)	1 (25)	0.712
Postration	8 (50)	5 (41.67)	3 (75)	0.248
Multiples convulsions	0 (0)	0 (0)	0 (0)	–
Respiratory failure	3 (18.75)	1 (8.33)	2 (50)	0.064
Shock	4 (25)	3 (25)	1 (25)	1
Jaundice	6 (37.50)	2 (16.67)	4 (100)	0.003
Bleeding	3 (18.75)	2 (16.67)	1 (25)	0.712
Analytical and radiological criteria				
≥2 criteria	7 (43.75)	3 (25)	4 (100)	0.009
Hypoglycaemia	2 (12.50)	1 (8.33)	1 (25)	0.383
Metabolic acidosis	4 (25)	1 (8.33)	3 (75)	0.008
Severe normocytic anaemia	0 (0)	0 (0)	0 (0)	–
Haemoglobinuria^a^	4 (26.67)	2 (16.67)	2 (66.67)	0.080
Hyperlactataemia^a^	2 (13.33)	0 (0)	2 (66.67)	0.002
Renal impairment	6 (37.50)	2 (16.67)	4 (100)	0.003
Pulmonary oedema	3 (18.75)	1 (8.33)	2 (50)	0.064
Parasitological data				
Parasitaemia (%)	12.5 (3.95–32.5)	7.3 (3.85–19.50)	46.5 (34–57)	0.008
Parasitaemia >30 %	4 (25)	1 (8.33)	3 (75)	0.008
Hyperparasitaemia	15 (93.75)	11 (91.67)	4 (100)	0.551

Categorical variables are described by counts and percentages; continuous variables are expressed as median (interquartile range)

*RBC* red blood cell

^a^Data available only for 15 participants

The median age was 40 years old (IQR 33–50), with a high proportion of male patients (11/16, 68.75 %). Most patients were travellers who had arrived from sub-Saharan Africa (11/16, 68.70 %), followed by immigrants “visiting friends and relatives”—temporary visitors- (VFR) (3/16, 18.75 %). None of the travellers completed a full course of chemoprophylaxis. Two patients were residents in endemic countries travelling to Spain (2/16, 12.50 %).

Half of the cohort presented three or more severity criteria, the most frequent being hyperparasitaemia (93.75 %), prostration (50 %), renal impairment (37.50 %) and jaundice (37.50 %). The median time to seek for health advice was 5 days (range 2.50–11.70) since the onset of symptoms. The median initial parasitaemia was 12.5 % (range 3.95–32.50 %). Four patients (25 %) presented with an initial parasitaemia above 30 %.

Patients finally treated with RBC exchange had a higher median initial parasitaemia compared to those who were treated with IVA alone (46.50 vs 7.30 %) and presented with more severity criteria (Table 2).

### RBC exchange and outcome

Four patients (25 %) received RBC exchange as an adjunctive treatment to IVA. This technique was initiated a median of 15.45 h after the first dose of IVA (IQR 9.05–20.58). The median duration of the technique was 2 h (IQR 1.80–2), using a median of 12 units of packed RBCs (IQR 11–14) to replace a median of 3794 ml (IQR 2977–4343).

The procedure was well tolerated without haemodynamic or hydroelectrolytical complications. The median difference in haematocrit and platelet count before and after RBC exchange was 2 % and −13,500/mm^3^, respectively. The median difference in blood calcium and potassium before and after the procedure was 0.15 mg/dl and −0.2 mmol/l, respectively. All patients survived and none of them had long-term renal or neurological sequelae. Two cases of delayed haemolysis could be detected: they completely recovered and did not show more complications.

### Parasite clearance times

**Fig. 1:**
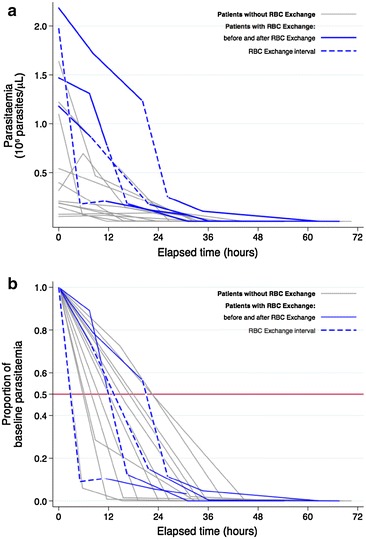
**a** Parasitaemia dynamics during the first 72 h under treatment with artesunate expressed as parasite load. **b** Parasitaemia dynamics expressed as the proportion of the baseline parasitaemia

The values of parasitaemia at each timepoint for each patient were analysed to estimate the PCT (Fig. 1). Table 3 summarizes the estimates from the mixed effects regression model including the effect of time on parasite clearance, thus obtaining the parasite elimination rate. The results of the model suggested that parasitaemia significantly decreased in relation with time. According to the estimated coefficient, the average reduction rate was 12 % per hour (95 % CI 10–14, p < 0.0001). Patients who received RBC exchange as an adjunctive therapy had a higher baseline parasitaemia compared to those who had IVA alone (p < 0.0001). When assessing effect modification by RBC exchange in the model, there was not any difference in the parasite elimination rate (coefficient for interaction 0.03, 95 % CI −0.02; 0.08, p = 0.2861).

**Table 3:** Mixed-effects regression model to estimate the changes in parasitaemia in 16 patients with severe malaria

Parameter	Coefficient	95 % confidence interval	p value
Log initial parasitaemia/µL^a^	12.02	11.24; 12.79	<0.0001
RBC exchange^b^	2.47	1.43; 3.51	<0.0001
Parasite elimination rate (/h)^c^	−0.13	−0.15; −0.10	<0.0001

*RBC* red blood cell

^a^Log initial parasitaemia for individuals who did not receive RBC exchange (intercept of the regression model)

^b^Difference in log initial parasitaemia for individuals who received RBC exchange vs those who did not

^c^Difference in log parasitaemia per unit increase of time (hour)

Based on the estimates of this model, the mean time to 50 % (PCT50), 90 % (PCT90), 95 % (PCT95) and 99 % (PCT99) parasite clearance were calculated (Table 4). PCT were calculated for the whole cohort without stratifying between those who had RBC exchange or not, since there was no evidence of an interaction between this therapy and time. Interestingly, 95 % of initial parasitaemia was cleared in a mean time of less than 24 h (21.60, 95 % CI 17.30–28.80), and almost the whole initial parasitaemia was cleared in less than 36 h (33.21, 95 % CI 26.60–44.30).

**Table 4:** Mean parasite clearance times estimated from the mixed-effects model in 16 patients with severe malaria

%	All patients (95 % CI) n = 16	Artesunate only (95 % CI) n = 12	Artesunate and RBC exchange (95 % CI) n = 4
PCT 50	5.0 (4.0–6.7)	5.0 (4.0–6.7)	6.3 (4.6–9.7)
PCT 75	10.0 (8.0–13.3)	10.0 (8.0–13.3)	12.6 (9.3–19.4)
PCT 90	16.6 (13.3–22.1)	16.6 (13.3–22.1)	20.9 (15.4–32.3)
PCT 95	21.6 (17.3–28.8)	21.6 (17.3–28.8)	27.2 (20.1–42.0)
PCT 99	33.2 (26.6–44.3)	33.2 (26.6–44.3)	41.7 (30.9–64.5)

Data expressed as hours

*PCT* parasite clearance time

## Discussion

In the present study, it is shown in the largest case series of severe imported malaria that use RBC exchange in a cohort treated with artesunate that IVA reduces quickly parasitaemia and that RBC exchange does not seem to add any benefit in terms of parasite clearance times.

The results of this retrospective follow-up study confirm the speed of action of IVA when used for imported severe falciparum malaria, reducing parasite clearance time in a similar way to other cohorts [5–7]. This case series, due to the mathematical population model applied, provides valuable data about parasite kinetics during the response to treatment. Due to the small sample size, the regression model was estimated assuming linear reduction of parasite density and, therefore, the real reduction could be underestimated at early times. The PCT included in this article can be used in the clinical practice by physicians treating imported severe malaria, as a reference for monitoring the microbiological answer to IVA.

Regarding the use of RBC exchange as an adjunctive therapy, it was found that it does not significantly contribute to parasite clearance [20]. However, this conclusion has to be taken very cautiously. First, small sample size and the study design may be responsible for this lack of evidence for the effect of the adjunctive therapy. Furthermore, the endpoint of the study was parasite clearance alone, which does not allow any firm conclusion for other biological plausible benefits of RBC exchange. Finally, the fact that the treatment protocol at this institution indicates RBC exchange for only selected patients may have reduced the effect modification of RBC exchange (selection bias).

In this study, a mixed effects model for log parasitaemia with fixed and random effects for initial parasitaemia, and fixed effects for exchange transfusion and time was used (Table 3). The authors were unable to demonstrate an effect of exchange transfusion on the decay of parasitaemia, as addition of an interaction between exchange transfusion and time was non-significant. This may have been due to the small sample size.

The development of safer apheresis therapies that can be used for severe malaria, such as RBC exchange, may have an impact on the medical management of the disease and there are many aspects of its use as adjunctive therapy susceptible of further investigation. One of the main questions about using RBC exchange for severe malaria together with IVA is which would be the accurate moment to initiate the apheresis. There is no debate that anti-malarial drug is the first step for this medical emergency. Its use is envisioned due to its good safety profile, confirmed by the data in this cohort, and its plausible biological benefits. One of the hypothetical concerns when using RBC exchange immediately after AVI is that the drug could be partially removed within the RBC exchange since artesunate has shown to have high binding to RBC [22].

Therefore, the use of RBC exchange should target to optimize IVA effect, not to replace it, not even diminish it. Since artesunate half-life is less than 15 min and 30–60 min for its metabolite dihydroartemisinin [23], the recommendation would be initiating RBC exchange not earlier than 3–4 h after initiating AVI. Due to its mechanism of action and its stage-specificity, artesunate generates more “once-parasitized” RBC than quinine when used in cases of severe malaria [24]. These RBC are erythrocytes whose parasite has been removed, by the spleen or by artesunate, becoming less mobile, spherical and particulate cell. The “pitting” effect explains disparity between haematocrit decrease and parasite count decay, as well as the rapid effect of artesunate [25]. One of the hypotheses is that once-parasitized RBC are major contributors to the development of post-artesunate delayed haemolysis (PADH) [11]. In this cohort of patients, just two cases of delayed haemolytic anaemia were detected, with no relation to RBC exchange. The authors hypothesize whether RBC exchange may prevent delayed anaemia with IVA.

## Conclusions

This case series illustrates the experience with the use of AVI for treating severe malaria, and the use of RBC exchange as adjunctive therapy. No serious adverse effects of RBC exchange transfusion were encountered in this small series. The study provides valuable data about technical procedure of RBC exchange and about monitoring microbiological response to artesunate. Although there remains limited evidence to support RBC exchange transfusion in severe malaria, it may be considered in special circumstances, such as the suspicion of artemisinin resistant hyperparasitemic falciparum malaria, the unavailability of IVA or in settings where parasite clearance may be prolonged, such as splenectomy.

A randomized clinical trial would provide some evidence about the prompt indication and initiation of RBC exchange when using artesunate, but it is currently unaffordable. Further multicentric, cohort studies should be performed to gradually increase the level of evidence to determine which selected patients with imported severe malaria may benefit from this adjunctive treatment and at which moment after artesunate administration will not reduce the drug’s benefit.
